# Quality Analysis of 3D Point Cloud Using Low-Cost Spherical Camera for Underpass Mapping

**DOI:** 10.3390/s24113534

**Published:** 2024-05-30

**Authors:** Sina Rezaei, Angelina Maier, Hossein Arefi

**Affiliations:** i3mainz, Institute for Spatial Information and Surveying Technology, School of Technology, Mainz University of Applied Sciences, D-55118 Mainz, Germany; rezaei.sina@hs-mainz.de (S.R.); angelina.maier@students.hs-mainz.de (A.M.)

**Keywords:** spherical camera, 3D point cloud, assessment, geometric features, relative orientation, data acquisition

## Abstract

Three-dimensional point cloud evaluation is used in photogrammetry to validate and assess the accuracy of data acquisition in order to generate various three-dimensional products. This paper determines the optimal accuracy and correctness of a 3D point cloud produced by a low-cost spherical camera in comparison to the 3D point cloud produced by laser scanner. The fisheye images were captured from a chessboard using a spherical camera, which was calibrated using the commercial Agisoft Metashape software (version 2.1). For this purpose, the results of different calibration methods are compared. In order to achieve data acquisition, multiple images were captured from the inside area of our case study structure (an underpass in Wiesbaden, Germany) in different configurations with the aim of optimal network design for camera location and orientation. The relative orientation was generated from multiple images obtained by removing the point cloud noise. For assessment purposes, the same scene was captured with a laser scanner to generate a metric comparison between the correspondence point cloud and the spherical one. The geometric features of both point clouds were analyzed for a complete geometric quality assessment. In conclusion, this study highlights the promising capabilities of low-cost spherical cameras for capturing and generating high-quality 3D point clouds by conducting a thorough analysis of the geometric features and accuracy assessments of the absolute and relative orientations of the generated clouds. This research demonstrated the applicability of spherical camera-based photogrammetry to challenging structures, such as underpasses with limited space for data acquisition, and achieved a 0.34 RMS re-projection error in the relative orientation step and a ground control point accuracy of nearly 1 mm. Compared to the laser scanner point cloud, the spherical point cloud reached an average distance of 0.05 m and acceptable geometric consistency.

## 1. Introduction

The significance of 3D point cloud quality assessment extends across various domains, including urban planning [1], environmental monitoring [2], archeology, cultural heritage documentation [3], and disaster management [4]. This detailed spatial information facilitates accurate terrain analysis, volumetric assessments, and object localization, facilitating informed processes [5]. Recent advancements in machine learning and computer vision techniques have further amplified the utility of 3D point cloud data, enabling advanced semantic segmentation, object detection, and classification tasks [6,7] based on the importance and diverse applications of 3D point clouds for their transformative impact on localization analysis [8,9]. This kind of dataset facilitates precise and comprehensive representations of three-dimensional (3D) spaces. Their importance stems from their ability to capture spatial information with high fidelity. In the past, the features and significance of 3D point clouds were restricted by only being provided by some sensors, such as LiDAR (Light Detection and Ranging), which is generally known for its remarkable detail and accuracy [10,11]. More recently, it has become possible to derive the clouds from optical cameras, e.g., DSLR (Digital Single-Lens Reflex) cameras and spherical cameras. Spherical cameras, as a tool for generating 3D point cloud data, have revolutionized spatial data acquisition due to their expanded field of view. These cameras mostly capture using an omnidirectional imagery system, enabling comprehensive scene representation and detailed 3D reconstructions. Recent advances in spherical camera technology have improved their accuracy, resolution, and efficiency. This has led to their increased use in a variety of applications [1]. Spherical cameras are used to produce 3D point cloud data for a wide range of applications, including virtual reality, urban planning, indoor mapping, and the preservation of cultural heritage [12]. Their ability to capture immersive visual data enables precise spatial analysis and visualization [1]. Additionally, point cloud datasets frequently contain supplementary attributes beyond spatial coordinates, such as color values, intensity, or classification information [13]. The richness of a dataset can be enhanced with these advanced analyses and interpretations. Incorporating color information can be used for texture mapping, while intensity data can provide information about surface or material properties [14]. Researchers have employed low-cost spherical cameras, commonly used in projects such as virtual reality and street view mapping, for three-dimensional reconstruction. The primary data of these cameras consist of fisheye images stored separately for each lens. These cameras adhere to dioptric geometry imagery systems [15]. Panoramic images can be generated by stitching together images from each lens of a camera to create overlapping images. This facilitates the implementation of three-dimensional reconstruction based on geometric principles. The volume of data acquisition is significantly reduced compared to other optical cameras, such as DSLR cameras.

Primary strategies for photogrammetric 3D reconstruction and data collection in confined spaces such as tunnels [16,17] and underpasses, or with restrictions on network design and camera placement [18], create critical challenges for proper data collection. On the other hand, generating robust 3D point clouds from image-based data requires a consistent and smooth level of lighting in the scene [19].

Generally, in the field of 3D point clouds, terrestrial laser scanning sensors or LSs (laser scanners) can provide data with acceptable accuracy [20] compared with other optical cameras. In addition, LSs have greater costs and more complex customization [21,22] compared to other optical cameras. Furthermore, the application of image-based algorithms and relative orientation under the cover of SFM-MVS (Structure From Motion—Multi-View Stereo) workflow has been increasingly used according to the capabilities and fast 3D modeling [23,24,25]. The workflow of an image-based algorithm begins with feature extraction from multiple images to establish their correspondence within the entire dataset. Camera position estimation is then performed before triangulation processing. This ultimately leads to the calculation of the 3D position of each image feature point (known as connection points) in a local system. This system is implemented through a series of computer vision algorithms [23]. These algorithms also solve calibration parameters through a self-calibration process and include various geometric image models, such as fisheye and equirectangular [17]. The results of the self-calibration process are compared to two separately performed calibrations to show the differences in accuracy. This allows low-cost optical camera consumers to use non-calibrated or calibrated images for 3D reconstruction and 3D point cloud generation. The precision and advantages of the relative and absolute orientation of 3D point clouds clarify the optimal network design and data capturing strategies for producing the final 3D point clouds according to the corresponding criteria. After this thorough analysis, the best 3D point cloud from the spherical camera can be compared with other types of sensors.

This paper evaluates the quality of a point cloud obtained from a graffiti underpass using fisheye and panoramic images from spherical cameras by using the principles of close-range photogrammetry in three-dimensional reconstruction. The goal is to analyze the 3D point cloud resulting from the optimized and most effective workflow among the proposed methods for data acquisition using various strategies. This optimization is based on relative orientation accuracy, the impact of image calibration methods, and network design. This sequential analysis of spherical camera use in point cloud generation can be compared with other sensors, both absolutely and uniquely. In terms of analysis, the evaluation of the geometric features of 3D point clouds is one of the most important analytical solutions for camera and fisheye lens images in order to achieve 3D reconstruction [16,23,26]. Therefore, this comparative analysis continues by measuring the distance between pairs of point clouds. This is calculated by two individual sensors and the geometric features of each point cloud are analyzed. This text describes the network design of camera stations and their orientations, data acquisition, and 3D reconstruction based on photogrammetry principles. This includes camera calibration and relative and absolute orientation, which result in the generation of 3D point clouds. Geometric analysis is also performed, including a mean and standard deviation assessment of spherical camera, laser scanner, and DSLR camera point clouds. The final section includes the conclusion, a discussion of the results, and statistical evaluations.

## 2. Materials and Methods

This study focuses on the use of spherical cameras as primary sensors for point cloud generation in the context of a graffiti underpass case study. The selection and evaluation of the most suitable spherical cameras was conducted based on their technical features. To evaluate the accuracy of the resulting spherical 3D point cloud, a laser scanner was used as a reference point cloud. Additionally, to compare the spherical camera point clouds with other optical sensors, a DSLR camera was also used. The network design and placement of imaging stations for spherical cameras were determined based on the principles of close-range photogrammetry. Pre-processing, including data organization, was performed on the acquired data before the point cloud was generated from the spherical camera images. This resulted in obtaining the desired results for processing and fisheye camera calibration of both the spherical and DSLR cameras. The photogrammetric reconstruction of the spherical camera images ultimately led to the generation of a three-dimensional point cloud from these images. The point cloud was evaluated in relation to the correspondence point clouds of the laser scanner and DSLR camera. The evaluation of the spherical camera point clouds was also based on the geometric features and the accuracies observed during the 3D reconstruction.

### 2.1. Case Study and Sensor Selection

**Figure 1:**
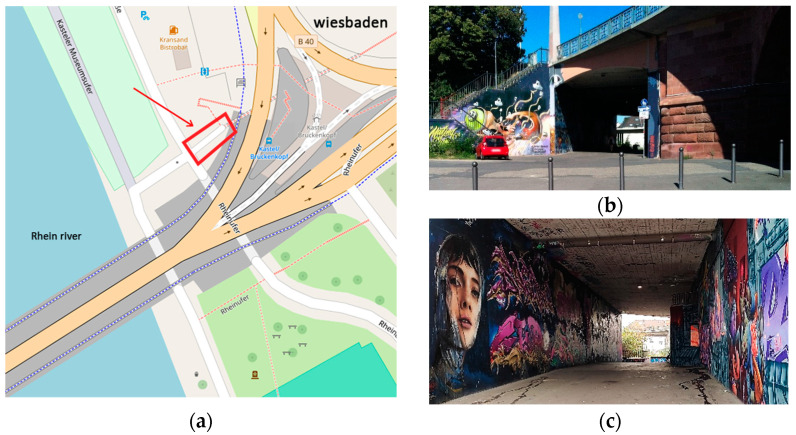
Location of the Theodor Heuss graffiti underpass (**a**); The underpass is placed in the German federal state of Hessen. The entrance (**b**) and the indoor environment (**c**) of the underpass.

The case study of this research was conducted in the graffiti underpass of the Theodor Heuss Bridge in Wiesbaden, Germany (Figure 1). The Theodor Heuss Bridge is a significant piece of infrastructure and connecting bridge between the Mainz and Wiesbaden cities in Germany. Beneath this bridge is an underpass with concrete walls painted with graphic drawings, and it was selected as the main structure in this research endeavor. The underpass is covered in graffiti and spans approximately 30 m in length, 4.5 m in height, and 6 m in width. It features an arched design with sloping ground and contains a single lighting resource, placed in the center of the ceiling, excepting outdoor lighting.

This paper excluded professional spherical cameras such as the Teledyne FLIR Ladybug, Professional360 Panono, Insta360 Pro2, and Weiss AG Civetta [23,24], due to their high price and more complex user interface for non-professional users, from the spherical cameras considered. Polydioptric spherical cameras are widely used due to their design principles, both for professional and low-cost sensors [24]. This is primarily due to two key factors. First, the technique provides full omnidirectional imaging, covering 360 degrees horizontally and 180 degrees vertically. The overlapping field of view facilitates image stitching to generate panorama images. Second, the imagery system has a higher resolution due to the use of stereo fisheye cameras simultaneously [23]. As a selection step, we aimed to determine the most suitable and cost-effective polydioptric spherical camera with the necessary features for data acquisition. These features include monitoring software, accessibility to raw files, high image resolution, and spatial resolution. Table 1 lists the specifications of the top four spherical camera brands.

**Figure 2:**
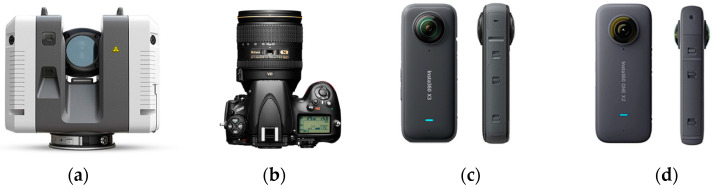
All sensors used in this study. (**a**) Leica RTC 360 laser scanner used as the reference sensor for point cloud quality assessment. (**b**) Nikon D800, 16 mm equivalent focal length for point cloud quality assessment. (**c**) Insta360 One X3, one of the best available low-cost spherical cameras (based on Table 1). (**d**) Insta360 One X2, one of the most optimized low-cost spherical cameras.

According to Table 1, the Insta360 One X2 and X3 cameras were used as the most optimized and best available spherical cameras based on their technical features in this research. The Insta360 One X3 has the best possible image resolution, but the Insta360 One X2 is more affordable and possesses optimized technical features in compare to other spherical cameras. To improve the 3D reconstruction and generation of 3D point clouds, an alternative sensor should be used alongside the spherical camera images as the base dataset. This sensor should assume the role of the most precise data collector for the graffiti underpass. Although LS surveying can be costly, it enables the precise and accurate detection of details, which is especially beneficial for reconstructing elements with complex shapes [27]. Accordingly, in this research, the Leica RTC 360 laser scanner (LS, Manufacturer: Leica Geosystems AG, Heerbrugg, Switzerland) was used to compare the quality (Figure 2). On the other hand, In order to make a comprehensive statement about the quality of the of the 3D point clouds, the data was captured by spherical cameras and other optical cameras, such as DSLR cameras, to compare the results. A well-known DSLR model (Nikon D800 with fisheye lens, 16 mm equivalent focal length, Manufacturer: Nikon Corporation, Tokyo, Japan) was used for data collection and comparison.

**Figure 3:**
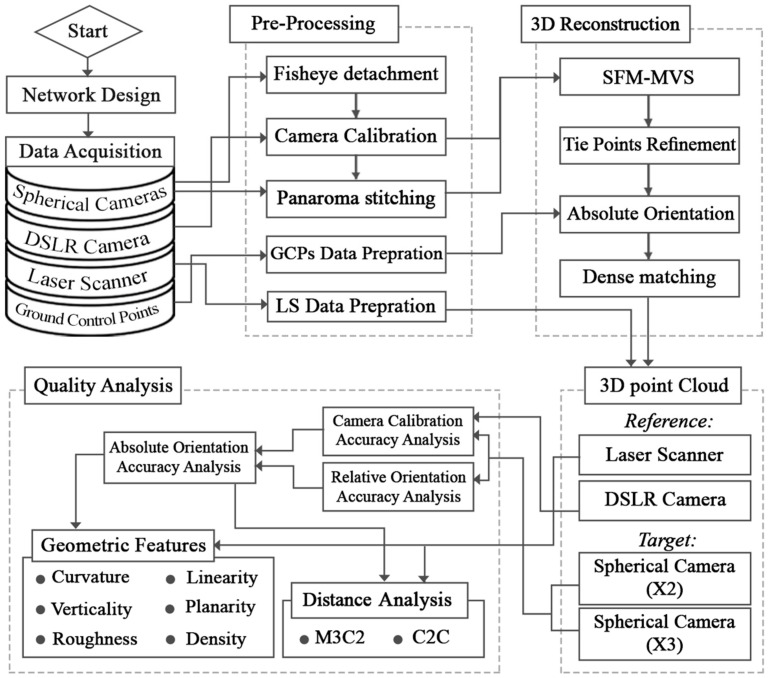
The flowchart of the present study in graffiti underpass point cloud analysis.

The following section (Figure 3) illustrates the proposed workflow and detailed structure of the spherical camera quality investigation. As it indicates, the main steps include network design, data acquisition, pre-processing, 3D reconstruction, and quality analysis, which are described in more detail in the following sub-sections.

In addition, several strategies were defined to find the best possible solutions for network design, appropriate data type, and calibration method when using spherical cameras (Table 2). The results in Section 3 will indicate the best solution in terms of relative and absolute orientation accuracy and also the accuracy of each calibration method. 

The network design options varied between stereo-mode and sideways-mode capture. There are two types of data in this framework, including fisheye and panorama images. In addition, there are three solutions for the calibration process, including chessboard calibration (calibration without splitting groups or in two separate chunks, and calibration with splitting groups or in one chunk) and the self-calibration method. The 8 stages of the process are all of critical importance for the generation of a point cloud. The resulting data also show impressive quality and accuracy. The further details of these stages will be explained in the corresponding sections.

### 2.2. Network Design

**Figure 4:**
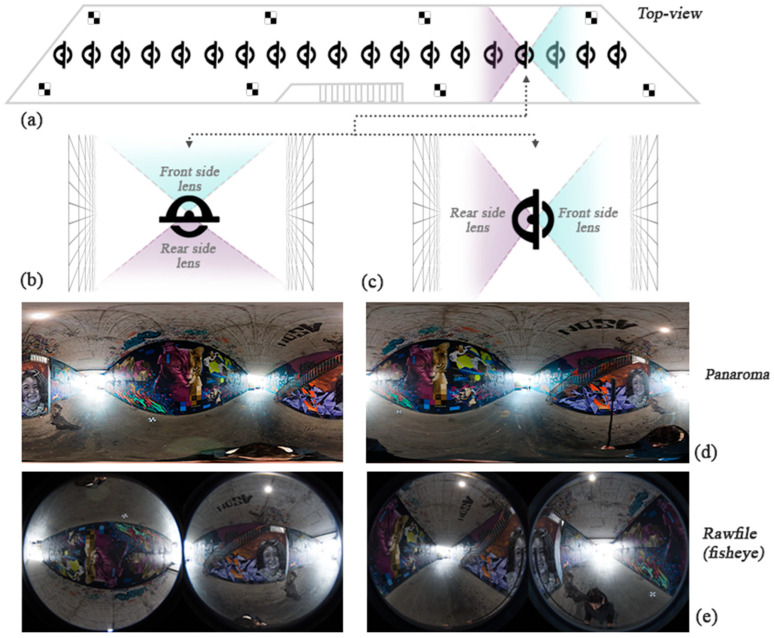
Two network design strategies were employed to achieve the highest possible accuracy of the 3D point clouds. In the graffiti underpass (top view: (**a**)), data capturing was repeated two times for each spherical camera with two different orientations: stereo mode (**b**) and sideways mode (**c**). Accordingly, the raw file images (**d**) and the panorama images (**e**) were exported for each implemented strategy.

**Figure 5:**
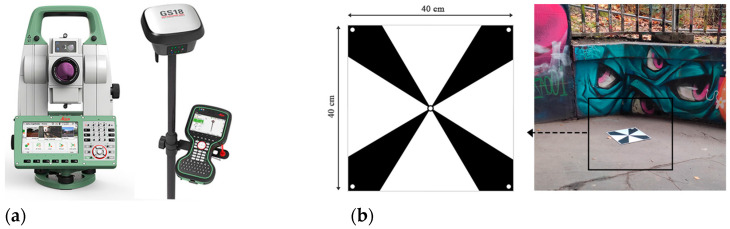
Total station and GNSS receiver used for defining the network of control points (**a**). The control point plates with 40 × 40 cm dimensions were big enough to be visible in the fisheye images of the spherical and DSLR cameras (**b**).

The optimal positioning of imaging stations requires the consideration of factors that determine the principles of network design and have significant effects on the outputs. The increase in geometric distortion towards the edges and corners of images, whether in fisheye or constructed panoramic images, magnifies the importance of feature placement in a less distorted camera field of view. Thus, determining the appropriate camera height and direction can minimize sequential reconstruction errors. As the height of the structure was about 6 m, the camera height was set to 2.7 m. Images were captured and positioned in two modes based on the geometry and corridor-shaped structure (Figure 4). In the first mode, the camera was directed towards the walls (sideways mode), the main features of the structure. In the second mode, the images were captured with the camera oriented towards the road of the underpass (stereo mode), and the space between each individual station was 1.5 m. In parallel, preparing for GNSS (global navigation satellite system) control point measurement is a crucial step to obtain the absolute positioning coordinates for the final reconstructed point cloud. Iron nails and control point plates were used to position the GPS points. This step was implemented directly with a total station without using the GNSS antenna. The points that indicated where to start and finish the capturing action were marked. Finally, eight targets were placed on the ground with adequate distribution to cover the entire ground section of the underpass. These targets provided control points later in the construction process. The image in Figure 5 displays one of the targets utilized.

### 2.3. Data Acquisition

The first step was to implement a GNSS measurement using a Leica GNSS antenna in order to obtain data correction information. Three points were placed near the tunnel outdoors. After measuring them, a prism was placed at the center of each control point to measure the geo-referenced coordinates. The control points were only located on the ground (due to the urban restriction on mounting control points on the underpass wall). 

After measuring the control points, the main step of data acquisition began with capturing images using both spherical cameras. The technical settings of the spherical cameras, specifically those pertaining to ISO and shutter speed (ISO value range: 100 to 250, shutter speed value range: 1/25 to 1/350), were automatically adjusted based on the lighting sensitivity. This was due to the lack of sufficient lighting conditions in the middle of the underpass. Each spherical camera, with an f/2 aperture and a focal length of 1.32 mm, captured 40 images (20 images in stereo mode and 20 images in sideways mode). Additionally, 54 photos were captured with the DSLR camera. In our DSLR data collection, only a specific section of the underpass was considered to account for quality comparison and not as a reference dataset. Therefore, the consistent value of ISO allowed for the capture of clear and detailed images with minimal graininess and noise (shutter speed value range: 1/13 to 1/160). Other settings included an ISO of 500, an aperture of F/4, and a focal length of 10.5 mm. In the final step, a reference measurement was taken using the Leica RTC 360 laser scanner from two equally distant viewpoints located at the entrance and exit of the underpass. The first laser scanner station was near the beginning of the underpass and the second one was almost at the end. The settings such as grid configuration, the quality of each scan, and double scan mode were checked. The scan time on both viewpoints was about 3 min.

### 2.4. Pre-Processing

#### 2.4.1. Data Organization

**Figure 6:**
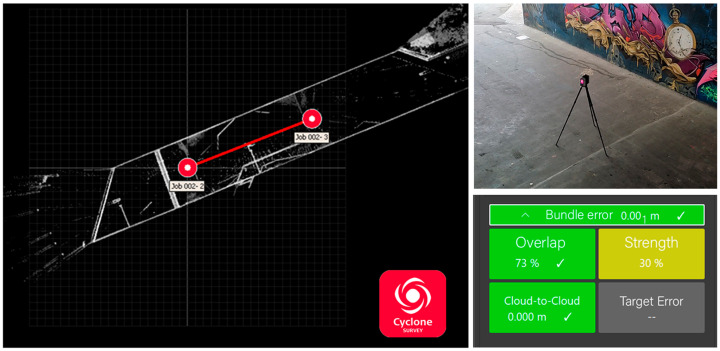
Laser scanner registration for two capturing stations in Cyclone Register 360 software (V2021.1.2); 73% overlapping area specified between two stations.

The workflow consisted of arranging the initial dataset for the further process and the related works of fisheye detachment, panorama stitching, and LS data preparation in the flowchart (Figure 3). It was necessary to import the laser scanner project file (.rtc360 file) into the Leica commercial software (Cyclone REGISTER 360, V2021.1.2) after data acquisition (Table 3). The significance of this step lies in the creation of an original file that can be converted into various adoptable point cloud formats, such as LAS or LAZ files. It can also be further processed in other software such as Cloud Compare (v2.12 beta). Initially, the accuracy of each scan was controlled by overlapping checks between the stations in Cyclone. Consequently, PTX or PTS files (raw files of LS) were converted into LAS files in Point Zip [28], an open-source program. Hence, the final applicable version of the laser scanner point cloud was generated for further processes in the assessment stage (Figure 6). To stitch panoramas from the raw dataset of the spherical cameras, we utilized the commercial software Insta360 Studio (v5.1.10). This allowed us to obtain both fisheye and panorama images. While fisheye images could be extracted directly from the raw file, the front- and rear-side images of the camera had to be separated and rotated to a realistic viewpoint in Adobe Photoshop (2019, v20.0.10). This was necessary to ensure a suitable input configuration for the subsequent 3D reconstruction software. Therefore, the pictures captured with the X2 and X3 camera were separated into those with a front and rear view. For the camera calibration process (Section 2.5.2), random images with different orientations and positions were captured. The goal was to cover the entire possible chessboard area in the defined FOV (field of view) with respect to the chessboard (for spherical and DSLR cameras).

#### 2.4.2. Camera Calibration

Two main solutions for fisheye camera calibration are considered in this step: the chessboard camera calibration method and the self-calibration method. The chessboard camera calibration method, known as Zhang’s method (1998), utilizes planar chessboard images in various orientations to determine the camera’s geometric model and lens distortion behavior through the calculation of camera calibration parameters. It assumes that the intrinsic parameters are constant during the relative orientation. On the other hand, the extrinsic parameters vary for each image in relative orientation. This process minimizes the re-projection error between the observed and predicted corner locations using a nonlinear optimization algorithm [6]. The accuracy and applicability of the imported calibration parameters depends on the quality of the source project photos, the camera stability, and the distance to the objects of interest [5]. The process of camera calibration, which is performed simultaneously with object identification during photogrammetric evaluation, is referred to as the self-calibration workflow. The images were automatically matched using the SfM algorithm and the calibration parameters were estimated through photogrammetric bundle adjustment during self-calibration [25]. 

**Figure 7:**
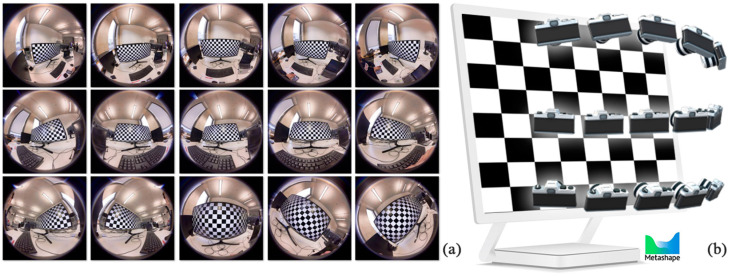
The process of chessboard camera calibration involves the use of datasets (**a**) and camera orientation (**b**), which are repeated for both the Insta360 One X3 and DSLR cameras (fisheye and normal lenses).

Thus, 15 images were captured with specific orientations from the chessboard using the X2, X3, and DSLR cameras. The spherical cameras’ raw fisheye images were used for capturing. To estimate the unique intrinsic parameters, it was important to capture the chessboard using the separate front-side and rear-side images of each spherical lens. This calibration framework is implemented in the professional commercial Agisoft Metashape software. It is the most commonly used commercial software due to its reliable reconstruction and a speedy and compatible user interface [8]. The front-side chessboard images of the Insta360 One X2 were imported into the software, and during the image processing operation, every single corner in the chessboard image was used for the modeling of the image distortion (Figure 7). There are 11 estimated parameters stored in an .xml file, ordered in a suitable format for the upcoming reconstruction process (listed in Table 4). In the following steps, the calibration file was imported into the Metashape project file, where the relative orientation of the main dataset was performed. The calibration process was repeated for the backside images one at a time, resulting in a different set of estimated parameters (Table 4). 

All of the parameters were checked as the consistent intrinsic parameters during the relative orientation calculation in order to obtain the stable relative orientation in the local 3D reference.

### 2.5. 3D Reconstruction

#### 2.5.1. SFM-MVS

As mentioned in Section 2.4.2, in this study, the main software used for 3D reconstruction and point cloud generation from the optical camera images was Agisoft Metashape.v2.1. The reconstruction process was conducted on a specific PC (GPU device: NVIDIA GeForce 930Mx—3 computing units @901 MHz, 2047 MB, CUDA, Mainz, Germany). The first step was to import the processed images into the software. As the second step, the relative orientation was obtained through the complete SFM-MVS workflow described in [1]. Key points and tie points were consequently identified on every image and matched between the overlapping parts of the images. The camera locations were estimated and finally the sparse 3D point cloud (with the alignment settings shown in Table 5) was successfully generated. The software used the Brown camera calibration model introduced in [7] with all eleven fisheye camera parameters during the alignment process. The alignment was set to high-accuracy mode, in which the software worked with the original sizes of the photos [7]. The imported calibrated parameters from two different solutions were used to determine the optimal relative orientation results. The first solution was to create a project file (called “chunk” in the software) corresponding to each lens and calibration file. Therefore, the 3D point cloud from each lens’s images was reconstructed separately. After the dense matching step, it was possible to join the chunks (related to the front-side images and rear-side images) together based on the ground control points and the camera position (in fact, every corresponding front-side image and rear-side image was captured in the same position). The second solution, however, was to import all the front-side and rear-side images of each camera in a single chunk and use the Metashape splitting camera option to join each image to the corresponding calibration file. Therefore, the 3D point cloud was reconstructed simultaneously for the front-side and rear-side images. As a matter of fact, all of these two-step solutions were used for each sensor (spherical and DSLR cameras). As the last solution, the SFM-MVS workflow was implemented without using pre-defined parameters, which is referred to as the self-calibration step. In this situation, Metashape solved the calibration parameters during the relative orientation which were no longer consistent during the alignment. In the next step, multiple tie point refinement solutions were carried out to achieve a more accurate sparse point cloud.

#### 2.5.2. Tie Point Refinement

**Figure 8:**
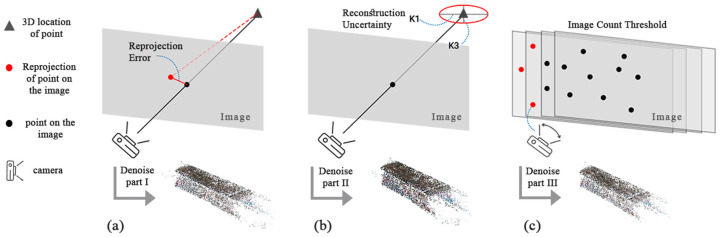
Three methods used for noise filtering from the reconstructed 3D point clouds: re-projection error (**a**), reconstruction uncertainty (**b**), and image count (**c**) threshold. For each step, point clouds were reduced by a certain amount.

To ensure the final sparse point cloud had minimal errors, it was necessary to remove outliers during the tie point refinement process. Metashape reconstructed all visible points that appeared in at least two photos. However, points that were visible on only two photos may have had poor accuracy. “Image count” filtering removed the unreliable points (Figure 8). The next criteria pertain to the maximum re-projection error. This was calculated in normalized units for all images where tie points were measured. The next solution was reconstruction uncertainty, which is the ratio of the largest semi-axis to the smallest semi-axis of the error ellipse of the triangulated 3D point coordinates [29]. The error ellipse corresponds to the uncertainty of the point triangulation alone without taking into account the propagation of uncertainties from the interior and exterior orientation parameters. The ideal setting for these parameters was based on the trial-and-error experiences mentioned in Table 6. This workflow was repeated for both spherical and DSLR data processing.

**Figure 9:**
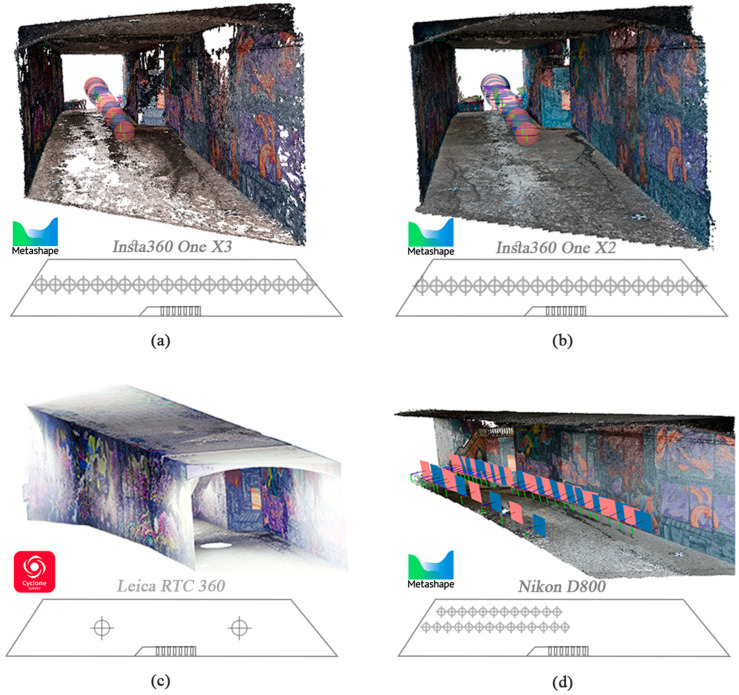
The network design is illustrated on the reconstructed 3D point cloud, accompanied by the relevant software: X3 camera with 20 stations (**a**), X2 camera with 20 stations (**b**), and DSLR camera with 38 stations (**d**), all implemented in Agisoft Metashape software to generate 3D point clouds. The RTC 360 laser scanner with two stations (**c**) performed the last process of this workflow.

After the refinement process, the final 3D point cloud of each sensor (Figure 9) was ready to compare and evaluate in the next step. 

#### 2.5.3. Absolute Orientation

During the absolute orientation step, which involved bundle adjustment optimization and geo-referencing [29], all ground control points were imported into the software. Their corresponding locations were automatically marked on each image. The coordinate system and relative orientation of the sparse point clouds were updated using the provided control points. Three of the control points were unchecked based on having the least number of projections on images, which was used as a criterion for determining the correctness of the sparse point clouds (known as check points). Simultaneously, the coordinates were set on an ETRS89 UTM 32N (European Terrestrial Reference System 1989) verified with the control points projected on Open Street Map (Figure 1a). Finally, the points from the 2D images were projected onto a 3D point cloud using a dense matching algorithm [30]. 

#### 2.5.4. Dense Matching

This dense point cloud process was based on the MVS algorithm [7]. The software was set to high-quality mode for dense matching and the final results were calculated based on the initial tie points. This workflow was repeated for both the spherical and DSLR datasets. The final output was a .txt file with the 3D point cloud coordinates.

### 2.6. Quality Analysis

#### 2.6.1. Camera Calibration Accuracy Analysis

To evaluate the accuracy of the calibration (Section 2.5.2), the RMS residual (with units in pixels) was used in each calibration solution to represent the re-projection errors for the tie points. These points were detected on the source images and averaged across all the images of the calibration group. The values corresponding to each item are listed in Table 7.

#### 2.6.2. Relative and Absolute Orientation Accuracy Analysis

For comparing the accuracy and quality of the relative orientation, tie point and point cloud numbers and RMS re-projection were linked to determine the best solution and result. Each corresponding value was exported in an individual report by Agisoft Metashape and is listed in Table 7. Similarly, for absolute orientation, two criteria were considered: the RMS error of control points (or GCPs accuracy) and the RMS error of control points (or control point accuracy) to also compare the quality (Table 8).

#### 2.6.3. Distance Analysis

The point clouds were compared based on their corresponding distances using two different methods. The first method is the cloud-to-cloud distance measurement (called C2C). It describes an essential step in aligning and comparing two point clouds. This process aims to register two or more point clouds with each other to ensure their spatial correspondence. The second method is the M3C2 (multiscale model-to-model cloud comparison) algorithm, in which the point clouds are viewed as surface models. The distances between these models are calculated using approximate levels. Compared to the M3C2 algorithm, the cloud-to-cloud comparison is a coarser comparison method that requires less computational effort. The M3C2 algorithm, on the other hand, provides a detailed analysis based on the smallest changes in the surface of the point cloud. These two analyses were carried out for the two spherical camera models and the laser scanner. 

Initially, the point clouds were imported into Cloud Compare software (v2.12 beta) for point cloud analysis. It was important that the data were in the same coordinate system or could be mapped onto it using suitable transformations. In the comparison process, manual control point selection was carried out. Initially, one of the point clouds was selected as a reference (LS). This served as the basis for aligning the other point clouds. The Cloud Compare C2C toolbox leverages common algorithms to calculate an initial estimation of the transformation between the reference and target point clouds. The calculated initial transformation was refined by the equivalent control points to achieve the optimal match of the point clouds. Between the reference and the target point clouds, the direct distance to the nearest neighbor was calculated and iterative methods were also used to minimize the deviations between the points. 

The M3C2 algorithm calculates distance by measuring the distance along a local normal vector estimated from each point’s neighborhood. This approach takes into account the local surface orientation in the distance computations. To find the locally averaged change between the two clouds, the algorithm projects search cylinders along the local normal vectors, therefore avoiding the need for meshing or gridding, which can introduce geometry errors in the terrain model [31]. The distance between the point clouds was determined within a cylinder defined on the reference point cloud, the center of which formed the normal of the average position of the points. The diameter of the cylinder was important to ensure the higher resolution of the result. If the cylinder diameter was too large, the average position of the points was determined from a larger surface [32]. This process was repeated for the pairs of point clouds: LS with X2, LS with X3, and X3 with X2.

#### 2.6.4. Geometric Features

In this case study, the color and intensity values of point clouds were not used in the geometric analysis. This is particularly important due to significant lighting effects at the start and end of the underpass, which can result in unreliable intensity values [9]. The geometric features of the point cloud were utilized to determine the geometric consistency and structure of the spherical point cloud in relation to the corresponding reference point cloud. The geometric features of the point clouds were defined based on certain elements of the covariance tensor ( $\Sigma_{i}$ ) [13].

(1) $$\Sigma_{i}=\frac{1}{N}\sum_{n\in p_{i}^{N}}\left(p_{i}-\bar{P}\right){\left(p_{i}-\bar{P}\right)}^{T}$$

where N is the number of adjacent points of $p_{i}$ , and $\bar{P}$ is the center of the adjacent [33]. These elements consist of the eigenvectors ( $e_{1}$ , $e_{2}$ , $e_{3}$ ) and eigenvalues ( $\lambda_{1}$ , $\lambda_{2}$ , $\lambda_{3}$ ) of the covariance tensor. The combination of these parameters led to the definition of the unique geometric features of point clouds of all sensors used, imported separately in Cloud Compare. These features included curvature, density, verticality, linearity, planarity, and roughness, defined within a neighborhood with respect to each point which relies on the sampling distance. The radius (r) for the neighboring points ( $p_{i}$ ) was determined in the Cloud Compare toolbox and the following results show their present effect on the spherical point clouds. the proper geometric structure was also shown in the laser scanner reference. The mentioned geometric features in this context were calculated with the following equations (Equations (2)–(7)) [30,31,34].

(2) $$\mathrm{Verticality}:\;1-p_{i}$$

(3) $$\mathrm{Curvature}:\;\frac{\lambda_{1}}{\lambda_{1}+\lambda_{2}+\lambda_{3}}$$

(4) $$\mathrm{Roughness}:\;p_{i}-\mu$$

(5) $$\mathrm{Linearity}:\;\frac{\lambda_{1}-\lambda_{2}}{\lambda_{1}}$$

(6) $$\mathrm{Planarity}:\;\frac{\lambda_{2}-\lambda_{3}}{\lambda_{1}}$$

(7) $$\mathrm{Density}:\;\frac{N}{\left(\frac{4}{3}\times \pi \times r^{3}\right)}$$

Each of these geometric features is associated with a specific behavior of a three-dimensional point cloud within the context of the geometric concept. Verticality measures the alignment of points with the vertical axis [33], curvature indicates the change in direction of the surface normal at a point, roughness is defined as the distance between the least-squares best-fitting plane and its neighborhood points contained in the radius (r) [35], and linearity determines how well points fit into a line, which is crucial for edge and feature extraction [34]. Planarity measures how points fit into a plane and is key for modeling surfaces [34], and density refers to the number of points within a given volume and affects the detail captured in the point cloud.

## 3. Results

In the pre-processing step, the following settings were implemented on the final output of the laser scanner point cloud in Cyclone (Figure 6) and for that of the commercial spherical cameras. Insta360 Studio stitched two fisheye images into a single panorama image with 6080 × 3040 resolution but an unknown camera geometric model (Figure 4). The spatial accuracy of the measured control points was 0.002 m.

The purpose of the described method in Section 2.2 for network design is to find out the most optimized solution for camera location and orientation. Otherwise, the final generated point cloud cannot cover the desired sections with sufficient accuracy and correctness. Twenty spherical images were captured two times in 2 min using both sideways mode and stereo mode. The optimized results of the network design can be derived from the relative orientation accuracy, data collection speed, and number of images. Since the data collection specifications were almost the same in both network design strategies, only relative orientation was evaluated. Also, as a result of Section 2.4, the stitching image processing of panoramic images and the camera calibration solutions were performed using three different strategies. Table 9 depicts the quantities of each accuracy value achieved by the process mentioned in Section 2.5. The relative orientation accuracy is described using re-projection accuracy, which refers to the difference between the observed tie points and the calculated corresponding ones defined by the root mean square error. 

**Figure 10:**
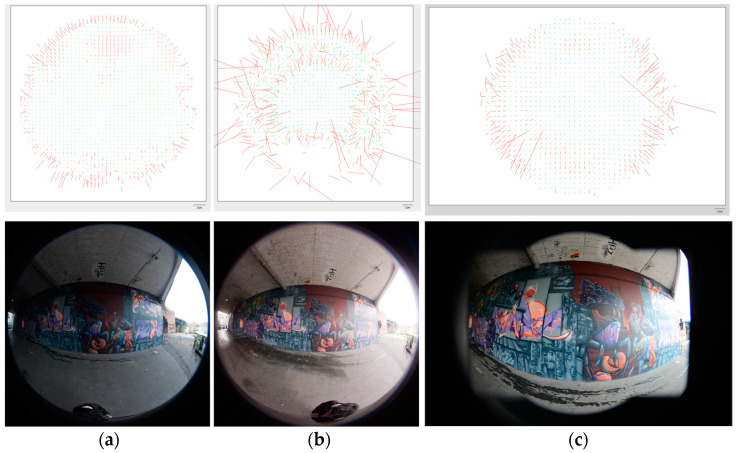
Distortion plots of calibrated fisheye lenses of spherical cameras (front side of X2 (**a**) and front side of X3 (**b**)) in comparison with fisheye lens of DSLR camera (**c**).

According to Table 7 and Table 9, the sideways-mode capturing strategy showed more reliable tie points and final point clouds compared to the stereo-mode strategy. Panorama images were better with respect to the RMS re-projection error compared to fisheye images. However, for the X2 camera, the best results were obtained in the sideways-capture mode without chessboard calibration, and slightly better results were shown for the tie points and point clouds. Among all three solutions for camera calibration, self-calibration during the alignment achieved more accurate RMS re-projection and calibration residuals. The DSLR camera was also used in order to compare these sequential results. The accuracy of calibration for the DSLR camera (0.158 RMS residual pixel error) was close to that of the X2 camera (0.427 RMS residual pixel error), but still better since the calibration method was genuinely equal. As shown in Figure 10, X2 camera distortion was even better than that of the DSLR fisheye lens in some parts around the border and basically all over the image plane. The X3 camera showed much more distortion (0.835 RMS residual pixel error) in comparison.

The absolute orientation for geo-referencing the 3D model was also measured by the ground control points and check points. The average accuracy of the GCPs was 0.02 m, and that of the check points, which indicates the correctness of the geo-referenced 3D point cloud, was 0.1 m (Table 10). If there was any insufficiently overlapping area, this shows that inaccurate tie points were generated [13]. Therefore, a higher number of DSLR images should be captured in comparison to a spherical camera dataset with a full field of view. The final 3D point cloud of the DSLR camera covers a small part of the underpass only for calibration and geometric accuracy assessments.

**Figure 11:**
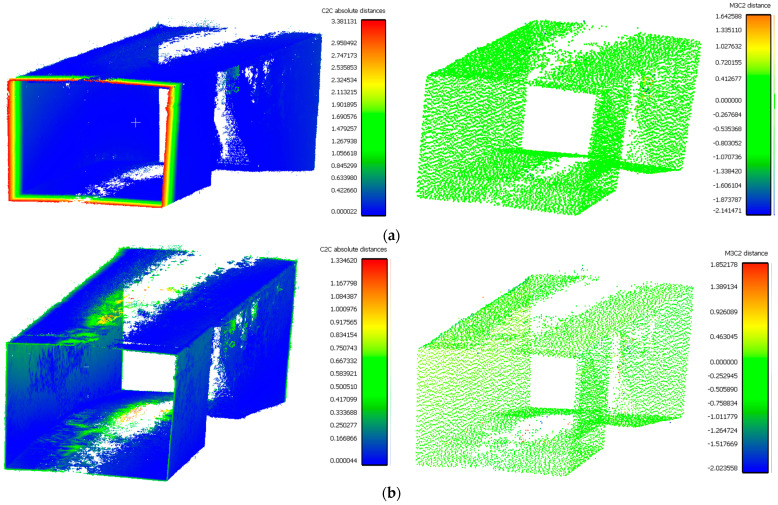

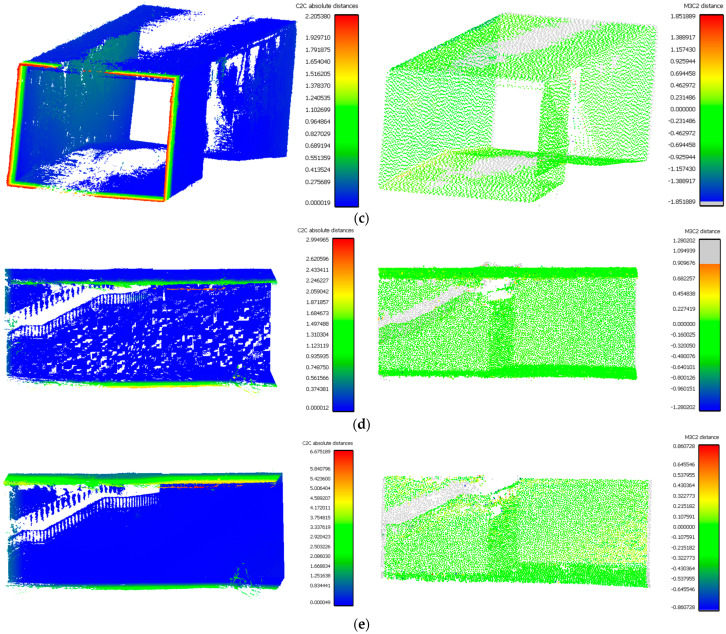
C2C distance models between each pair of point clouds: LS compared with X2 (**a**); X3 compared with X2 (**b**); X3 compared with LS (**c**); X2 compared with DSLR (**d**); X3 compared with DSLR (**e**).

The distance measurements between the reference point cloud and each spherical point cloud, and the mean distance and standard deviation between two point clouds, are shown in the table below (Table 11 and Figure 11). The M3C2 mean distance for the X3 camera was nearly 5 cm compared to 3 cm for the X2 camera. The C2C values were 22 cm and 18 cm for X3 and X2. Since the LS point cloud was four times denser than the spherical ones (about 262 million points in the LS cloud compared to 52 million points in the X3 camera cloud), this indicates that there are some occluded areas which created a significant gap in the generation of spherical 3D point clouds.

The geometric rendered outputs are depicted according to the following features in Table 12. In order to have the possibility of comparison between two individual models with the same geometric feature, a consistent radius of 0.02 is assumed for the filter window size to calculate and include the neighboring points. According to relative orientation accuracy, this attitude yielded the best results. Therefore, some geometric features were identified in comparison only for these elite point clouds of X2 and X3 spherical cameras (based on Table 11).

Due to limited data acquisition with the DSLR camera, the geometric model of the 3D point cloud only depicts one side of the underpass wall. Based on the roughness function, which indicates the noise level of the point clouds, the wall camera model in the X3 model had more noise points compared to the X2 model. Generally, when the lens was positioned towards the walls, it was expected that the density of these areas would be much higher with respect to the overhead ceiling and ground. Nevertheless, the X2 camera model achieved a much denser point cloud than the X3 camera (Table 11, b). Among the features of a structure, surfaces are essential elements. The geometric features of surface curvature, in terms of other geometric features such as verticality, are more prominent in the X2 model compared to the X3 one. Some geometric features, such as verticality, are independent of network design and only vary based on the type of sensor which creates the point cloud. According to the results, there is a significant connection among geometric features in terms of accuracy. For example, by increasing the roughness or noise level of the point cloud, other geometric features will become dramatically unstable through the entire model.

## 4. Discussion and Conclusions

**Figure 12:**
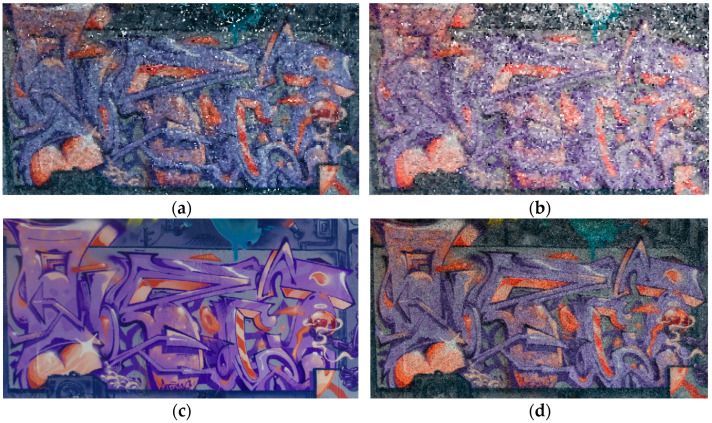
Quality of colorized 3D point clouds generated by spherical cameras X2 (**a**) and X3 (**b**) in comparison with LS (**c**) and DSLR (**d**).

This study presents a comprehensive workflow for 3D point cloud quality analysis using low-cost spherical cameras. It covers the entire process from initial data acquisition to the generation of 3D point results through a photogrammetric framework. The strategies are divided into four solutions for network design and three for camera calibration. In network design, for both models of spherical cameras, the sideways mode was the best solution based on the relative orientation accuracy and completeness of the point cloud. In this orientation, the color of the point cloud was more realistic and precise according to the LS reference point clouds (Figure 12).

Vividly, the colorized 3D point clouds generated by the spherical cameras were less precise and realistic than that generated by the LS cameras. Despite this comparison, LS also generated better results than DSLR. Due to the expansion of lighting and rebalancing the exposure through the entire field of view, spherical camera images generally cannot capture the true color values in restricted areas with limited lighting such as underpasses.

In camera calibration, split mode calibration, where the relative orientation of the image from both lenses is generated by a single part of the processing software, produces the best result in absolute orientation. However, for overall relative orientation accuracy, self-calibration during the SFM-MVS algorithms based on the Brown fisheye calibration model (Section 2.5.2) achieved the best result. The respected Elite 3D spherical point cloud also used this calibration. In contrast, the number of tie points in the dense point cloud dramatically increased with this solution. The use of checkerboard calibration accounts for the fact that there are different distortions for the front and back lenses in each spherical camera, although this fact was considered as an assumption before starting the data collection. In camera calibration, distortion dramatically increases in the borders of the fisheye images. All 11 parameters of the Brown calibration model were used to model distortions for the corresponding fisheye lenses in the spherical cameras. Strikingly, the fisheye lens of the DSLR camera showed less distortion with respect to spherical camera lenses, which had an intense impact on the generated 3D point cloud noise level. Since chessboard calibration in this framework and toolbox did not refine the distortions during image alignment despite the use of calibration parameters, the final 3D point cloud inevitably contained significant noise. After the noise reduction, considerable numbers of points were interactively removed from the initial sparse point cloud. So far, the best relative orientation was achieved with a 0.1263 RMS re-projection error and 9601 reliable tie points as the best result. For control points with control point implementation, the accuracy and correctness were simultaneously achieved in return for the absolute orientation of the local 3D point cloud in the ETRS89/UTM zone 32N coordinate system. 

**Figure 13:**
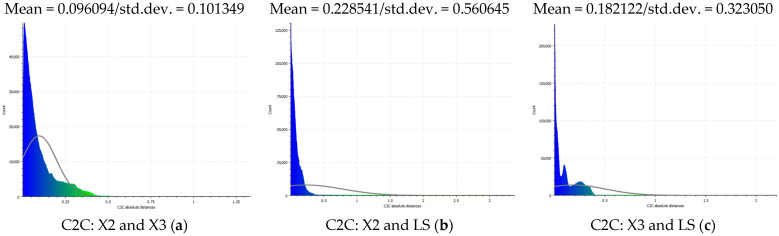
Mean and standard deviation of distance per point (with C2C solution) between X2 and X3 (**a**), X2 and LS (**b**), and X3 and LS (**c**) cameras in the following Gaussian histogram plots. The values and corresponding colors of plots are mentioned in Table 11.

**Figure 14:**
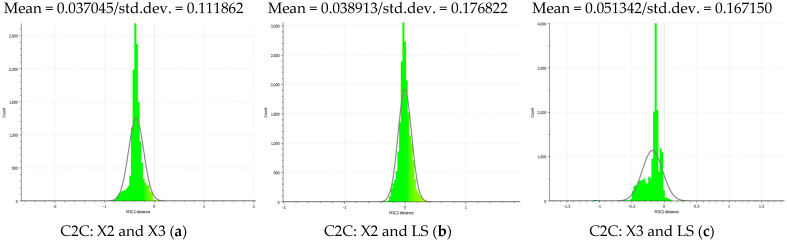
Mean and standard deviation of distance using normal vectors (with M3C2 solution) between X2 and LS (**a**), X3 and LS (**b**), and X2 and X3 (**c**) cameras in the following Gaussian histogram plots. The values and corresponding colors of plots are mentioned in Table 11.

The best 3D point cloud of each spherical camera was compared to the two reference point clouds of the LS and DSLR cameras. This was based on geometric features and distance evaluation. For the X3 camera, the compared values when calibrating by splitting groups into two chunks were 0.05 m each for the GCP and check point accuracy. The calibration method with just one chunk achieved values of 0.02 and 0.04 m. This shows that this calibration method of the side-captured images provides the more accurate results. The quality of the check points was also better than the method in which images were taken sideways. In the Cloud Comparison process, the corresponding values of the Elite Sphere 3D point cloud can be read using colored areas on the scale during C2C measurements. The goal is to highlight the accuracy of the point cloud. Achieving as many blue areas as possible is crucial, as these areas show virtually no deviation from the reference point cloud. However, the areas with very large deviations are shown in red. For further analysis, the same parameters were adopted for the M3C2 algorithm. Taking the LS and X2 sphere cameras as an example, the mean distance and resulting standard deviation are shown in the figures below (Figure 13 and Figure 14). As mentioned in Section 2.6.3, each 3D model used standard deviation and mean distance as measurement criteria. Therefore, they were used in Gaussian statistical representation to detect the deviation.

The distance measurements show the higher accuracy of the X2 camera in terms of correctness and lower noise based on standard deviation. The higher average distance of the C2C method is essentially due to some significant gaps in the spherical point cloud compared to the LS method. This difference has a direct impact on the average distance value for this distance calculation. On the other hand, by considering the normal vectors of the M3C2 algorithm, more accurate mean and deviation measurements can be achieved. According to the geometric features, all the mentioned features (Table 12) of the spherical models are slightly weaker than those of the LS model, except for the density, due to the noisy point cloud. The main reason for this can be attributed to the challenging part of the spherical point cloud, where the middle part of the ceiling and floor is generated. Due to the lack of sufficient overlapping images and the smaller number of connection points, a large gap is seen, and in the edges, the inconsistency of geometric features is clearly visible. Regardless of these gaps, only the noise level of the spherical clouds made a difference, and at a certain point, e.g., in terms of density, spherical point clouds achieved a better or denser result compared to the DSLR model. Geometrically, it is also important that the structure of the spherical model is correct, but due to the noise and gap area in the ceiling and floor, they play the main role in the differences between the spherical and LS models. In conclusion, the 3D point cloud analysis workflow has demonstrated that spherical models are reliable in terms of accepted accuracy and correctness at short range by applying the optimal data acquisition strategies such as network design and leveraging the SFM-MVS photogrammetry method. Due to the fast data acquisition, small data volume and low initial cost of the instruments, two of the best low-cost spherical cameras (Table 1) were considered to be acceptable optical cameras for 3D point cloud generation. The Insta360 One X2 had a slightly more stable geometric image model compared to the Insta360 One X3. In contrast to the X2 camera, this model offers extensive functions and diverse applications. These conclusions are the result of a comparison between the results, using the DSLR and LS models as reference models. In addition, according to Table 2 and the following results in Section 3, the most optimal strategy for network design based on the accuracy and quality of the 3D point cloud was the side capture image solution. It also includes fisheye images and self-calibration as data types. Ultimately, uniform illumination remains a key challenge for spherical cameras.

The present study does not constitute a comprehensive examination of outdoor structures in 3D reconstruction using low-cost spherical cameras and their associated challenges in reconstruction and data acquisition, which ultimately result in the generation of a 3D point cloud. However, this can be expanded in future works by considering structures that exhibit more complex geometrical characteristics. The calibration of spherical cameras requires consistent definitions and a reliable geometric model for this kind of optical camera. A suitable solution for camera calibration can effectively impact any photogrammetric results, including 3D point clouds and other corresponding products. Further assessments of noise reduction levels could also be insightful for this field. Processing through commercial software restricts researchers to developing and examining detail calculations. Therefore, approaches to modular processing solutions will be more comprehensive and reliable.

## Figures and Tables

**Table 1:** The specification of well-known spherical cameras. Noticeably, the technical features of Insta360 spherical cameras are more suitable for data acquisition process [1,9,10,11].

Camera Model	Brand	Resolution (Megapixel)	Image Resolution (pix)	Weight (kg)	Raw File Accessibility	Cost (USD)	Monitoring Software
Gear360	Samsung	15	4096 × 2048	0.13	✓	369	✓
Theta Z1	Ricoh	23	6720 × 3360	0.18	×	559	×
Theta X	Ricoh	60	11,008 × 5504	0.17	✓	796	×
Max360	GoPro	16	5760 × 2880	0.16	✓	399	✓
One X	Insta360	18	5760 × 2880	0.11	×	259	✓
One X2	Insta360	48	6080 × 3040	0.14	✓	290	✓
One X3	Insta360	18.72	11,968 × 5984	0.18	✓	399	✓

**Table 2:** Different strategies of data acquisition and pre-processing implemented in this study. Panorama images used no calibration solution at this stage.

Solutions	Network Design Solution	Data Type Solution	Calibration Solution
Solution 1	Sideways mode	Fisheye images	Self-calibration
Solution 2	Sideways mode	Fisheye images	Chessboard calibration (splitting mode)
Solution 3	Sideways mode	Fisheye images	Chessboard calibration (without splitting mode)
Solution 4	Sideways mode	Panorama images	---
Solution 5	Stereo mode	Fisheye images	Self-calibration
Solution 6	Stereo mode	Fisheye images	Chessboard calibration (splitting mode)
Solution 7	Stereo mode	Fisheye images	Chessboard calibration (without splitting mode)
Solution 8	Stereo mode	Panorama images	---

**Table 3:** Detailed specifications of the laser scanner and the captured dataset.

Laser Scanner Accuracy Settings	Values
3D point accuracy	1.9 mm in 10 m|2.9 mm in 20 m|5.3 mm in 40 m
Ground/space sampling resolution	3 mm (highest sampling resolution)
Angular accuracy of LS	18″
Range accuracy	1 mm in 10 ppm
Optical camera for colorized point cloud	3 cameras, 36 MP resolution
Noise distance	0.4 mm in 10 m|0.5 mm in 20 m

**Table 4:** The calibration parameters for spherical and DSLR sensor units are defined in pixels for all values.

Calibration Parameters	X3 (Front Lens)	X3 (Rear Lens)	X2 (Front Lens)	X2 (Rear Lens)	D800
F (Focal length, pix)	1554.8544	1555.5011	848.4453	848.1613	2150.0099
cx (principal point offset in X axis, pix)	−2.4490	−1.0948	−8.2083	−0.4822	−14.8078
cy (principal point offset in Y axis, pix)	20.4959	24.6478	3.7923	−0.2146	−3.1103
K1 (radial distortion, pix)	0.0726	0.0893	0.0515	0.0465	−0.0357
K2 (radial distortion, pix)	0.0017	−0.0424	−0.0103	0.0018	−0.0124
K3 (radial distortion, pix)	−0.0246	0.0230	0.0052	−0.0058	0.0316
K4 (radial distortion, pix)	0.0107	−0.0067	−0.0042	−0.0003	−0.0236
B1 (non-orthogonality, pix)	−0.5308	−0.7564	−0.8797	−0.8279	3.2830
B2 (non-orthogonality, pix)	1.2574	1.7960	0.0991	0.0945	0
P1 (tangential distortion, pix)	−0.0007	−0.0007	4.8196 × 10^−5^	−0.0002	−0.0001
P2 (tangential distortion, pix)	−0.0007	−0.0016	−0.0005	−0.0006	−0.0010

**Table 5:** Image alignment settings in Agisoft Metashape software.

Alignment Settings	Values
Tie point threshold	8000
Key point threshold	40,000
Camera calibration methods	Two methods: Brown fisheye model (self-calibration)|Zhang’s method (chessboard calibration)
Accuracy mode	High|Generic preselection

**Table 6:** The experimental values for the threshold definition of noise filtering.

Calibration Parameters	Reconstruction Uncertainty	Re-Projection Error	Image Count
Threshold value	30	~1	2

**Table 7:** Comparing the results of the Insta360 spherical cameras’ (X2 and X3) fisheye images for different camera calibration and network design solutions. The optimal results underlined.

Sensor	Camera Orientation	RMSE Re-Projection	Calibration RMS Residual (pix)	Number of Point Clouds	Number of Tie Points
	Without pre-calibration (refined during relative orientation–self-calibration)				
X2	stereo	0.136758	0.427	4,588,728	16,077/67,275
X3	stereo	0.126319	0.835	13,875,434	9601/54,016
X2	sideways	0.766283	1.377	18,676,576	43,782/62,556
X3	sideways	0.343216	0.742	30,471,652	21,968/118,537
	Calibration without splitting groups (in two separate chunks)				
X2	stereo	0.176492	0.694	5,292,014	14.210/67.275
X3	stereo	0.200214	3.1	14,747,707	6850/54,014
X2	sideways	0.26795	1.28	14,242,671	17,238/62,431
X3	sideways	0.4989	3.762	39,937,968	37,810/58,581
	Calibration with splitting groups (in one chunk)				
X2	stereo	0.27507	1.364	6,388,632	26,857/54,185
X3	stereo	0.37041	1.226	10,652,336	5514/57,984
X2	sideways	0.24312	1.083	14,118,170	35,312/57,520
X3	sideways	0.32965	2.357	37,484,289	32,946/56,378

**Table 8:** Comparing the results of the DSLR Nikon D800 camera with a fisheye lens for different relative orientation and camera calibration solutions.

Calibration	RMSE Re-Projection	Calibration RMS Residual (pix)	Number of Point Clouds	Number of Tie Points
Not calibrated	0.165979	0.158	42,193,069	14,306/29,616
Calibrated	0.32821	1.56	11,542,445	15,136/29,614

**Table 9:** Comparing the results of the Insta360 spherical cameras’ (X2 and X3) panorama images for different network design solutions.

Sensor	Camera Orientation	RMSE Re-Projection	Number of Point Clouds	Number of Tie Points
X2	stereo	0.702747	9,489,270	19,518/57,564
X3	stereo	0.268801	17,671,659	21,288/42,700
X2	sideways	0.282473	11,542,445	28,341/37,765
X3	sideways	0.414831	40,166,750	6456/26,155

**Table 10:** Comparing Insta360 spherical cameras’ (X2 and X3) 3D point clouds in absolute orientation accuracy using the below criteria. The optimal results underlined.

Sensor	Camera Orientation	GCP Accuracy (m)	Check Point Accuracy (m)
without calibration (being refined during relative orientation)			
X2	stereo	0.003592	0.047857
X3	stereo	0.027332	0.100306
X2	sideways	0.102562	0.7241114
X3	sideways	0.001714	0.010127
calibration without splitting groups (in two separate chunks)			
X2	stereo	0.068918	0.368897
X3	stereo	0.056098	0.504710
X2	sideways	0.066433	0.453532
X3	sideways	0.045880	0.079698
calibration with splitting groups (in one chunk)			
X2	stereo	0.01	0.06
X3	stereo	0.02	0.13
X2	sideways	0.01	0.07
X3	sideways	0.04	0.13

**Table 11:** Distance measurement between each pair point clouds for LS and the X2 and X3 spherical cameras.

Compared Clouds	C2C Distance Measurement		M3C2 Distance Measurement	
	Std. Deviation	Mean Distance	Std. Deviation	Mean Distance
X2 and X3	0.101	0.096	0.176	0.038
X2 and LS	0.560	0.228	0.111	0.037
X3 and LS	0.323	0.181	0.167	0.051
X2 and DSLR	0.342	0.152	0.077	0.015
X3 and DSLR	1.171	0.91	0.083	0.007

**Table 12:** Geometric features of the X2, X3, DSLR camera, and laser scanner 3D point clouds. The radius was 0.02 for all the data types.

Geometric Features	Insta360 One X3	Insta360 One X2	Laser Scanner	DSLR Camera
Roughness	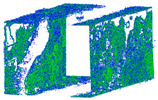	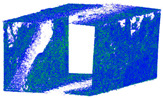	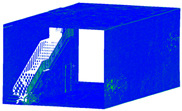	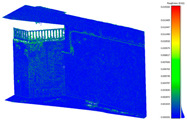
Density	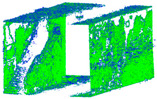	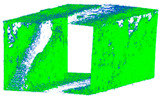	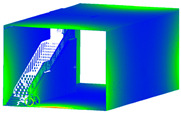	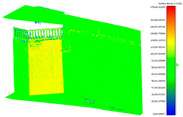
Verticality	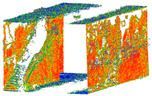	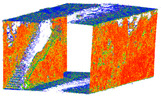	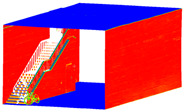	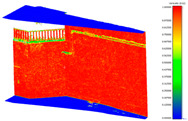
Planarity	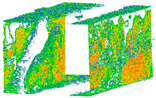	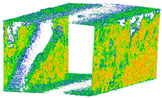	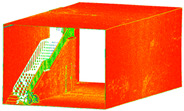	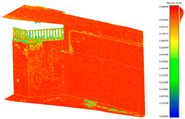
Linearity	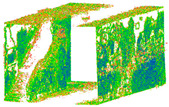	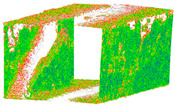	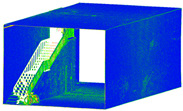	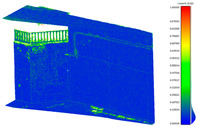
Curvature	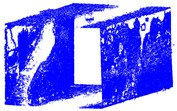	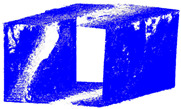	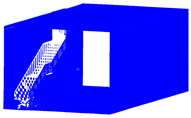	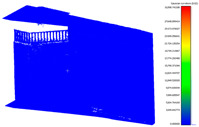

## Data Availability

Data are contained within the article.
