# Correction: Rezaei et al. Quality Analysis of 3D Point Cloud Using Low-Cost Spherical Camera for Underpass Mapping. *Sensors* 2024, *24*, 3534

**DOI:** 10.3390/s25103068

**Published:** 2025-05-13

**Authors:** Sina Rezaei, Angelina Maier, Hossein Arefi

**Affiliations:** i3mainz, Institute for Spatial Information and Surveying Technology, School of Technology, Mainz University of Applied Sciences, D-55118 Mainz, Germany; rezaei.sina@hs-mainz.de (S.R.); angelina.maier@students.hs-mainz.de (A.M.)

## Reference Correction

There was an error in the original publication [1]. Original ref. [1] has been retracted, therefore, ref. [1] has been replaced by original ref. [12], and the original citation of ref. [12] has been changed into [1]. With this correction, the order of some references has been adjusted accordingly. The authors state that the scientific conclusions are unaffected. This correction was approved by the Academic Editor. The original publication has also been updated.
